# Synthesis of hydrophilic and hydrophobic carbon quantum dots from waste of wine fermentation

**DOI:** 10.1098/rsos.170900

**Published:** 2017-12-20

**Authors:** Massimo Varisco, Denis Zufferey, Albert Ruggi, Yucheng Zhang, Rolf Erni, Olimpia Mamula

**Affiliations:** 1 HES-SO – University of Applied Sciences Western Switzerland, Haute Ecole d'Ingénierie et d'Architecture de Fribourg, Institute of Chemical Technology, 1705 Fribourg, Switzerland; 2 Department of Chemistry, UNIFR – University of Fribourg, 1700 Fribourg, Switzerland; 3 EMPA – Swiss Federal Laboratories for Materials Science and Technology, Electron Microscopy Center, 8600 Dübendorf, Switzerland

**Keywords:** carbon quantum dots, waste valorization, wine lees, luminescent material, quantum dots synthesis

## Abstract

Wine lees are one of the main residues formed in vast quantities during the fermentation of wine. While toxic when applied to plants and wetlands, it is a biodegradable material, and several alternatives have been proposed for its valorization as: dietary supplement in animal feed, source for various yeast extracts and bioconversion feedstock. The implementation of stricter environment protection regulations resulted in increasing costs for wineries as their treatment process constitutes an unavoidable and expensive step in wine production. We propose here an alternative method to reduce waste and add value to wine production by exploiting this rich carbon source and use it as a raw material for producing carbon quantum dots (CQDs). A complete synthetic pathway is discussed, comprising the carbonization of the starting material, the screening of the most suitable solvent for the extraction of CQDs from the carbonized mass and their hydrophobic or hydrophilic functionalization. CQDs synthesized with the reported procedure show a bright blue emission (*λ*_max_ = 433 ± 13 nm) when irradiated at 366 nm, which is strongly shifted when the wavelength is increased (e.g. emission at around 515 nm when excited at 460 nm). Yields and luminescent properties of CQDs, obtained with two different methods, namely microwave and ultrasound-based extraction, are discussed and compared. This study shows how easy a residue can be converted into an added-value material, thus not only reducing waste and saving costs for the wine-manufacturing industry but also providing a reliable, affordable and sustainable source for valuable materials.

## 1. Introduction

Wine lees are an unavoidable phytotoxic waste produced by wine fermentation and are becoming increasingly expensive to treat as stricter environment protection measures are implemented worldwide. Wine lees are composed of dead yeast, proteins, fibres, lipids, sugars and organic acids [1], and therefore offer a valuable source of organic matter. Many studies [2–6] have been pursued in order to recover products from this waste, but all of them focus on the extraction of specific compounds (e.g. yeast extracts, flavonoids, tartaric acid or fatty acids). However, in our approach, this waste is transformed into carbon quantum dots (CQDs). The different types of compounds that constitute the wine lees represent all the important reactants needed for the synthesis of CQDs: easily degradable organic compounds for the actual formation of CQD core (carbon sources). Furthermore, this waste also contains long-chain carboxylic acids that will not decompose during the high-temperature carbonization step and will act as passivating agents which increase the stability of the CQDs by preventing their agglomeration.

Carbon and carbon-based materials are known mostly as black, insoluble, non-emitting solids. On the other hand, CQDs are a relatively new class of fluorescent carbon-based materials, easily dispersible in water, discovered in 2004 by Xu *et al.* during their work on carbon nanotubes [7]. CQDs distinguish themselves from the other carbon-containing materials by their small size (less than 10 nm), their wide absorption and tuneable emission [8]. Since their discovery, the interest in CQDs grew exponentially [9,10] because of their numerous applications: catalysts (e.g. oxidation of benzoyl alcohol [11,12], degradation of methyl blue if combined with TiO_2_ or SiO_2_ [13], H_2_ generation by water splitting or CO_2_ reduction [14,15]), bio-imaging agents [16], photoluminescent [17,18] or electroluminescent materials [19], metal ion detectors [20–22] and electron acceptors for photovoltaics [23].

Furthermore, they are easily produced from almost any carbon source [24] (e.g. chemicals such as citric acid and ethylene diamine (EDA) or food waste such as brewing residue in the production of beer [25] or banana juice [26]). Different synthetic pathways (bottom-up approaches) [27,28] are known to produce CQDs: ultrasonication [29], treatment in acid [30], microwave [31], hydrothermal [32] or direct combustion [33]. Several top-down approaches are also known, e.g. laser ablation [34], electrochemical release [35], arc discharge [36] or plasma treatment [37]. Most of these approaches can yield CQDs in one-pot synthesis, but that has the disadvantage of losing the possibility to adapt the CQDs' features to specific applications. To overcome this limitation, researchers developed different functionalization strategies. Even if CQDs tend to be inert towards a vast range of environments, under the right conditions they can be easily functionalized [38] to further tune their properties (quantum yield (QY), potential, etc.) [39,40].

In this paper, we present CQDs synthetized by the combustion method [25], extracted from the black mass using ultrasounds or microwaves. After extraction, these CQDs were reacted with HNO_3_ to increase the number of carboxylic acid moieties. In the following step, the functionalization, the carboxylic groups are reacted with EDA [24] or dodecylamine (DDA) [38] to increase the QY or to change the range of solvents in which they can be dispersed, respectively.

## 2. Experimental

### 2.1. Materials

All chemicals were purchased from Sigma-Aldrich at the highest purity available and used without further purifications. Wine lees were Humagne Rouge from Valais, Switzerland and Chasselas from Vaud, Switzerland. The oven for the carbonization of wine lees was a Nabertherm muffle furnace working in air atmosphere; the ultrasonication apparatus was a Sonics & Materials VibraCell; and the microwave oven was a Biotage Initiator Classic. The distillation of dodecylamine was performed in a Büchi Glass Oven B-585. The ink was obtained by dispersing 0.02 g of R-4A in 100 ml of a UV curable varnish (HAPA AG), which was further deposited on a polystyrene sheet by ink-jet printing using a home-made printing platform.

### 2.2. Carbonization of the wine lees

The dried lees were carbonized for 3 h at 300°C under air conditions. The black residue was ground in an agate mortar for 5 min. The thus-obtained black powder is stable and could be stored at room temperature (RT) until further use. In the following, **W** is designing the samples obtained from white wine lees, while **R** is for those obtained from red wine lees.

### 2.3. Solvent screening for the extraction of carbon quantum dots

A total of 200 mg of the black powder was suspended in 20 ml of ethanol. The suspension was stirred for 3 days at room temperature. Then the solution was filtered with a filter paper and the filtrate was centrifuged at 4000 r.p.m. for 1 h. The solution was concentrated under reduced pressure, resulting in 120 mg (60%) of CQDs. The same extraction procedure was performed with various solvents: water (130 mg, 65%), methanol (111 mg, 55.5%), isopropanol (11 mg, 5.5%), ether (10 mg, 5%), toluene (2 mg, 1%), n-heptane (62 mg, 31%) and cyclohexane (14 mg, 7%).

### 2.4. Solvent extraction with an ultrasound stepped microtip (compounds **W-1A** and **R-1A**)

A total of 2 g of the black powder was suspended in 40 ml of ethanol and submitted to ultrasonication using a stepped microtip at full power (130 W) for 5 min. The mixture was filtered with a 0.22 µm filter and the solution was concentrated under reduced pressure to give 0.24 g (12%) of a dark solid.

### 2.5. Solvent extraction by microwave oven (compounds **W-1B** and **R-1B**)

To 500 mg of the black powder, 20 ml of ethanol was added. The mixture was heated in a microwave oven for 1 h at a constant pressure of 18 bar. The mixture was filtered with a 0.22 µm filter. The solution was concentrated under reduced pressure to obtain 134 mg (26.8%) of a dark solid.

### 2.6. Oxidation by acidic treatment (**W-2A**, **W-2B**, **W-2**, **R-2A** and **R-2B**)

The procedure of Liu *et al.* [30] was followed with minor modifications: to 812 mg of the CQDs obtained with one of the above-mentioned extraction methods (or the black powder obtained by the thermal treatment for **W-2**), 10 ml of nitric acid (5 M) was added. The mixture was refluxed for 4 h and cooled down to RT. Distillation to remove the excess of nitric acid afforded 786 mg (97%) of a yellowish/pale brown solid.

### 2.7. Functionalization with ethylenediamine (**W-3A**, **W-3B**, **W-3**, **R-3A** and **R-3B**)

The procedure of Cintya D'Angelis *et al.* [24] was followed with minor modifications: to 172 mg of the CQDs obtained after the acidic treatment (or obtained with one of the above-mentioned extraction methods or the black powder obtained by the thermal treatment for **W-3**), 10 ml of thionyl chloride was added. The reaction mixture was refluxed for 4 h. Excess of thionyl chloride was distilled off and EDA (5 ml, 92.5 mmol) was added to the residual mass. The mixture was heated at 115°C and stirred at this temperature for 4 h. Excess of EDA was distilled off. The solid obtained was dispersed in 10 ml of water and it was dried under reduced pressure. This procedure was repeated three times. The traces of EDA were finally distilled off by a glass oven (high vacuum, 80°C).

### 2.8. Functionalization with dodecylamine (**W-4A**, **W-4B**, **W-4**, **R-4A** and **R-4B**)

The procedure of Mei *et al.* [38] was followed with modifications: to 80 mg of the CQDs obtained after the acidic treatment (or obtained with one of the above-mentioned extraction methods or the black powder obtained by the thermal treatment for **W-4**), 10 ml of thionyl chloride was added. The reaction mixture was refluxed for 4 h. Excess of thionyl chloride was distilled off and a solution of DDA (1 g, 5.4 mmol) in 5 ml of toluene was added to the residual mass. The mixture was refluxed and stirred at this temperature for 4 h. Excess of DDA was distilled off by a glass oven (high vacuum, 80°C).

### 2.9. Characterization

Photoluminescence spectra were collected with a Perkin Elmer LS 50 B; QYs were measured with a Edinburgh FS 5 equipped with an integrating sphere; lifetimes were measured with a Edinburgh Lifespec II with a picosecond pulsed diode laser at 405 nm (20 MHz); and emission was filtered at 54.7° (magic angle) with a polarizer. Transmission electron microscopy (TEM) micrographs were collected on a FEI Tecnai Spirit and on a JEOL JEM2200fs; Fourier-transform infrared (FT-IR) spectra were collected on a Bruker ALPHA's Platinum ATR.

## 3. Results and discussion

### 3.1. General synthetic procedure

**Figure 1.**
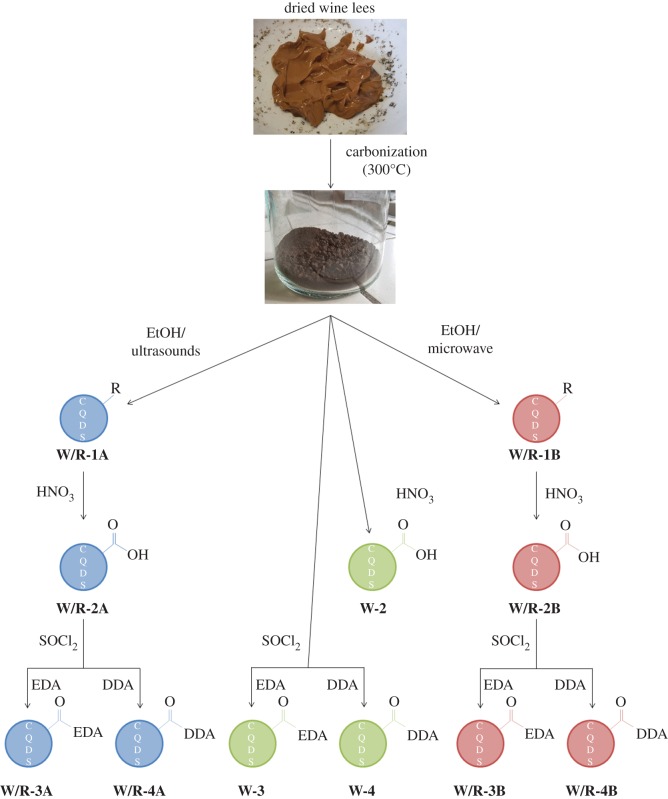
Schematic representation of the treatments carried out in this study.

The wine lees are an ideal starting material for the synthesis of CQDs. As already stated in the introduction, it contains both the carbon source and the passivating agent. Figure 1 shows an overview of the synthetic pathways followed. After the first step, a high-temperature reaction (oven, air atmosphere, 300°C), CQDs are indeed formed, but they are mixed with carbon residue, hence the need for an efficient extraction process. Two methods were chosen, namely ultrasonication or microwave-assisted extraction (**1**). Exploitable CQDs are obtained after this step, but to take full advantage of their features, further functionalizations are needed. To have an efficient functionalization, a reaction of the carboxylic acid moieties was chosen; hence an oxidation in HNO_3_ is performed to maximize the number of carboxylic acid functions present on the surface of the CQDs (**2**). To react these functions with amine-containing molecules, an intermediate, the acid chloride-functionalized CQDs are obtained by treatment with SOCl_2_. Different functionalizations can be performed at this point and, in this paper, we will present a reaction with EDA (**3**) that aims to increase the QYs of the CQDs and a reaction with DDA (**4**) that aims to obtain CQDs dispersible in apolar media.

In between all steps, mass spectra were recorded and no signal belonging to the reacted molecule could be observed; instead background noise was detected.

### 3.2. Preliminary study on solvents and concentrations

Extraction tests were performed by stirring 200 mg of carbonized lees with different solvents for 3 days, with the aim of determining the solvent with the best extraction capacity. The results of these experiments are reported in the electronic supplementary material, table S1. Water, ethanol and methanol are superior to the other solvents, with an average extraction yield of 60%. All other solvents give only poor yields (less than 10%), with the remarkable exception of n-heptane, which gives a 30% yield. However, the lack of fluorescence observed in the n-heptane solution after extraction suggests that the material extracted with this solvent does not contain CQDs.

The optimal concentration that affords the highest photoluminescence was determined experimentally (electronic supplementary material, figure S1). A solution of 0.2 g l^−1^ shows the highest photoemission. Furthermore, the wavelength of maximal emission is concentration-dependent. The highest emission for the 0.1 g l^−1^ solution is at 427 nm, while the maximum emission for the 0.4 g l^−1^ solution is slightly red-shifted to 434 nm.

Although water is a safe and green extraction solvent and is also used for CQD production [41], it did not produce the CQDs with the strongest emission (among the solutions with the same concentration). As ethanol gives the solutions with highest emission, (electronic supplementary material, figure S2), it was chosen as the extraction solvent for the next steps.

### 3.3. Extraction of carbon quantum dots from carbonized white wine lees

Although extraction by stirring the carbonized lees with ethanol gives encouraging results, the required long extraction time (3 days) makes this procedure unpractical. Ultrasonication- and microwave-assisted extractions are expected to shorten the extraction times in a fast and controlled fashion, thus enabling the development of a practical and reproducible extraction protocol. Carbonized white wine lees were suspended in ethanol and extracted by ultrasonication (**W-1A**) and by microwave (**W-1B**), respectively, for 5 min and for 1 h.

**Figure 2.**
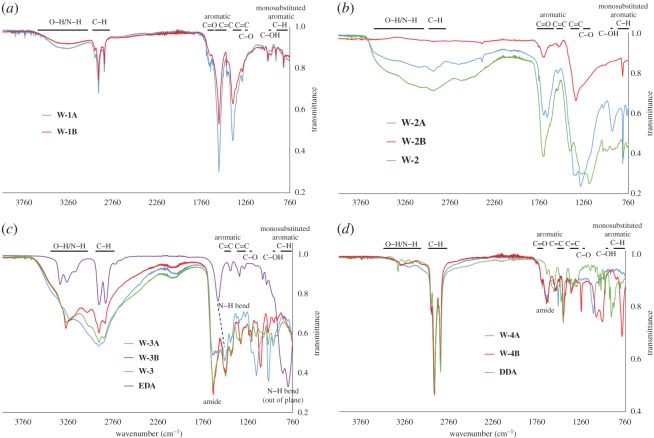
IR spectra of: (*a*) **W-1A** (blue line) and **W-1B** (red line); (*b*) **W-2A** (blue line), **W-2B** (red line) and **W-2** (green line); (*c*) **W-3A** (blue line), **W-3B** (red line), **W-3** (green line) and EDA as the reference (green line); (*d*) **W-4A** (blue line), **W-4B** (red line) and **DDA** as the reference (green line).

**W-1A** shows an emission maximum *λ*_max_ at 415 nm, while **W-1B** is slightly red-shifted with *λ*_max_ at 423 nm (electronic supplementary material, figure S3). Ultrasonication resulted in CQDs with a twofold higher QY with respect to microwave extraction (QY =  2.5% versus 1.2%, respectively), while microwave extraction gave almost a double yield with respect to ultrasonication (26.7% versus 12%, respectively). The CQDs prepared by the two methodologies were characterized by IR absorption spectroscopy and TEM microscopy. In spite of the different optical properties observed for **W-1A** and **W-1B**, both materials show very similar IR spectra (figure 2*a*), with medium C--C (1560 and 1400 cm^−1^), C--H (2915 and 2850 cm^−1^) stretching and weak carbonyl (1660 cm^−1^) stretching. This is in agreement with the expected signal of the long-chain carboxylic acid moieties which compose the passivation layer of the CQDs.

**Figure 3.**
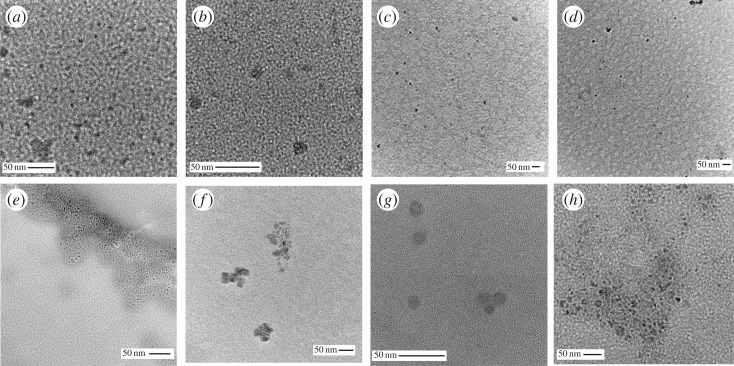
TEM micrographs of (*a*) **W-1A**, (*b*) **W-1B**, (*c*) **W-2A**, (*d*) **W-2B**, (*e*) **W-3A**, (*f*) **W-3B**, (*g*) **W-4A**, (*h*) **W**-**4B**. Scale bars, 50 nm.

Nevertheless, TEM microscopy shows that **W-1A** and **W-1B** possess significant morphological differences. In fact, CQDs extracted using ultrasonication (figure 3*a*) tend to be smaller than the ones obtained with the microwaves (10 nm versus 15 nm, figure 3*b*).

In both cases, most of the product has a circular shape, as expected for CQDs. Additionally, some aggregates have been observed for **W-1A**. It should be pointed out that this quick and affordable method easily provides ready-to-use CQDs, which can be further functionalized to improve their optical properties and tune their solubility in polar or apolar solvents.

### 3.4. Oxidation by acidic treatment

Optical and solubility properties of CQDs can be finely tuned upon functionalization with suitable organic molecules. Thus, in order to have an efficient functionalization with amine-containing molecules, the number of carboxylic acids on the surface of the CQDs should be increased (e.g. by treatment with HNO_3_).

Indeed, IR spectra indicate that this reaction took place (figure 2*b*). The strongest signals previously seen belonging to the C–C and C–H bonds (2915, 2850, 1560 and 1400 cm^−1^) become much weaker in comparison to the vibrations of the C–O (carbonyl) and C–O (alcohol) bonds (1700, 1285 and 940 cm^−1^). The spectrum recorded for the CQDs obtained by direct acid reaction on the carbonized lees (**W-2**) is comparable to that for **W-2**A. **W-3B** shows a slightly different spectrum. Even if the typical bands of the carboxylic acid moieties (see above) are broad, the signals belonging to the C–H bonds (2915 and 2850 cm^−1^) show much weaker intensity, meaning that the reaction was successful.

Furthermore, the morphology was studied by TEM and the micrographs are shown in figure 3*c*,*d*: the cores of the CQDs were not influenced by the treatment. Compared to the **W-1** series, smaller aggregates (average size less than 20 nm for both **W-2A** and **W-2B**) could be observed.

Finally, the photoluminescence decreased considerably, affording CQDs with QY equal to 0.4% for **W-2A** and 0.8% for **W-2B** (photoluminescence spectra in the electronic supplementary material, figure S3), indicating that the long-chain carboxylic acids indeed acted as passivating agents (thus increasing the photoluminescence for the **W-1** series) [18] and after the oxidation/hydrolysis, the long-chain functionalities were removed. The gap between the *λ*_max_ is increased: 415 nm for **W-2A** and 438 nm for **W-2B**.

### 3.5. Functionalization with ethylenediamine

To further improve the QY of the CQDs and to keep the hydrophilicity unaltered, EDA was chosen as the functionalization agent. After the treatment with HNO_3_, CQDs were reacted with SOCl_2_ in order to form the acid chlorides. These intermediates, more active than their carboxylic precursors, form amides by reaction with the amine-terminated molecules.

To prove the successful functionalization with EDA, IR spectra were recorded (figure 2*c*). The signals belonging to C–H stretching of the ethyl moiety at 2850 and 2920 cm^−1^ are almost unaffected by this reaction. As expected, the N–H bending (out of plane) at 810 and 870 cm^−1^ almost disappeared, while the N–H stretching (3275 and 3350 cm^−1^) decreased in intensity. Moreover, the signal of the carbonyl stretch previously observed at 1700 cm^−1^, corresponding to carboxylic acids, is slightly shifted to 1650 cm^−1^ as expected for amide groups, and the signal of the N–H bending at 1590 cm^−1^ is shifted to 1510 cm^−1^. Furthermore, TEM micrographs (shown in figure 3*e* and *f*) prove that the core of the CQDs was not much affected by all these treatments. In addition, **W-3B** shows only dots in the form of aggregates (bigger than 50 nm), whereas **W-3A** shows dots with the usual shape and size (approx. 10 nm), confirming that the core was not influenced by the treatments.

**Figure 4.**
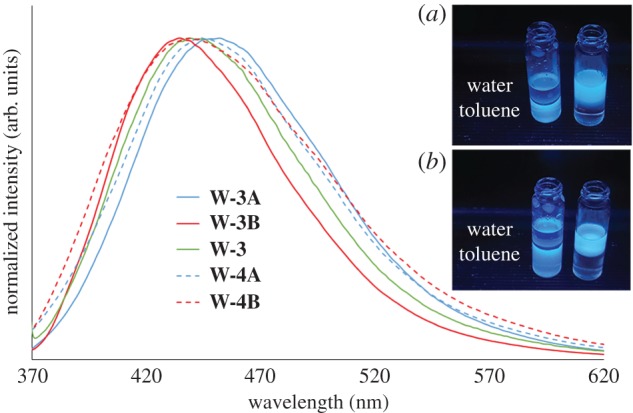
Emission spectra (excitation at 360** **nm, concentration 0.2** **g l^−1^ in deionized water for the **W-3** series and concentration 0.2** **g l^−1^ in toluene for the **W-4** series) of **W-3A** (solid blue line), **W-3B** (solid red line), **W-3** (solid green line), **W-4A** (dashed blue line) and **W-4B** (dashed red line). In the inset are shown pictures of the following samples, under excitation at 366 nm: (*a*) **W-4A** dispersed in toluene (left) and **W-3A** dispersed in water (right); (*b*) **W-4B** dispersed in toluene (left) and **W-3B** dispersed in water (right).

To prove the importance of post-extraction functionalization, the emission of **W-3A** and **W-3B** was compared to the results of the directly functionalized, non-extracted, carbonized lees (**W-3**), as shown in figure 4. CQDs functionalized with EDA possess the highest photoemission among all our samples. **W-3A** shows a slightly red-shifted emission (452 nm) when compared with **W-3B** (435 nm) and W-3 (439 nm). QYs and lifetime values were recorded (table 1). The QY values obtained (around 6%) are close to the literature values, even if some studies mention much higher results [42,43].

**Table 1.** Quantum yields, decay times, average lifetimes, radiative rate constants *k*_r_ and non-radiative rate constants *k*_nr_ of all samples.

samples	quantum yield (%)	first decay time *τ*1 [ns]	second decay time *τ*2 [ns]	average lifetime 〈*τ*〉 [ns]	radiative rate constant *k*_r_ [s^−1^]	non-radiative rate constant *k*_nr_ [s^−1^]
**W-1A**	2.53	2.76 (28.0%)	7.90 (72.0%)	6.46	3.91 × 10^4^	1.51 × 10^6^
**W-1B**	1.16	2.71 (29.8%)	8.29 (70.2%)	6.63	1.75 × 10^4^	1.49 × 10^6^
**W-2A**	0.41	2.08 (33.5%)	7.63 (66.5%)	5.77	7.10 × 10^3^	1.73 × 10^6^
**W-2B**	0.85	2.11 (35.9%)	7.72 (64.1%)	5.71	1.49 × 10^4^	1.74 × 10^6^
**W-3A**	6.80	1.73 (34.4%)	7.54 (65.6%)	5.54	1.23 × 10^5^	1.68 × 10^6^
**W-3B**	6.30	1.74 (29.5%)	7.40 (70.5%)	5.73	1.10 × 10^5^	1.64 × 10^6^
**W-4A**	5.41	2.68 (21.7%)	10.65 (78.3%)	8.92	6.06 × 10^4^	1.06 × 10^6^
**W-4B**	4.43	2.46 (34.5%)	9.00 (67.5%)	6.79	6.53 × 10^4^	1.41 × 10^6^
**R-1A**	1.94	2.88 (31.6%)	8.34 (68.4%)	6.61	2.93 × 10^4^	1.48 × 10^6^
**R-1B**	1.81	2.71 (30.1%)	8.21 (69.6%)	6.55	2.76 × 10^4^	1.50 × 10^6^
**R-2A**	1.00	2.41 (37.6%)	8.29 (62.4%)	6.08	1.65 × 10^4^	1.63 × 10^6^
**R-2B**	0.42	2.21 (36.0%)	7.95 (64.0%)	5.89	7.14 × 10^3^	1.69 × 10^6^
**R-3A**	5.50	1.76 (31.5%)	7.59 (68.5%)	5.75	9.56 × 10^4^	1.64 × 10^6^
**R-3B**	6.50	1.79 (29.2%)	7.04 (70.8%)	5.51	1.18 × 10^5^	1.70 × 10^6^
**R-4A**	3.59	2.47 (28.7%)	9.40 (71.3%)	7.41	4.85 × 10^4^	1.30 × 10^6^
**R-4B**	4.35	2.51 (34.1%)	8.95 (65.9%)	6.75	6.44 × 10^4^	1.42 × 10^6^

The QYs are very similar, as well as both components of the exponential decay. At the same concentration, **W-3B** is preferable because it is obtained in a slightly higher yield and it has a higher absorption than **W-3A** (electronic supplementary material, figure S4), resulting in a stronger emission (similar QY). The average decay time 〈*τ*〉 for **W-3A** and **W-3B** is 5.54 ns and 5.73 ns, respectively. These results are in the same range as the common literature values [44]. Combining these values with the QYs, it is possible to determine the radiative (*k*_r_) and non-radiative (*k*_nr_) rate constants. In our case, we obtain a *k*_r_ of 1.23 × 10^5^ s^−1^ and a *k*_nr_ of 1.68 × 10^6^ s^−1^ for **W-3A**. A *k*_r_ of 9.56 × 10^4^ s^−1^ and a *k*_nr_ of 1.64 × 10^6^ s^−1^ are obtained for **W-3B**. The influence of the non-radiative contribution is more than one order of magnitude higher than that of the radiative one, explaining why the QYs are so low.

### 3.6. Functionalization with dodecylamine

To broaden the application field, a functionalization with hydrophobic moieties DDA was performed in order to prepare CQDs dispersible in organic solvents (hydrophobic). The same procedure as for EDA involving SOCl_2_ was applied before reacting the DDA with the dots.

To prove that DDA is attached to the CQDs, IR spectra were recorded and are shown in figure 2*d*. The strong C-H signals (at around 2900 cm^−1^) are clearly dominant for both **W-4A** and **W-4B** and the other DDA signals are also visible (e.g. at 1560, 1460 and 720 cm^−1^).

Furthermore, the strong carbonyl stretching appears at 1650 cm^−1^, which is characteristic of an amide group. As observed for the **W-3** series, the N-H bending (out of plane), at around 900 cm^−1^, disappeared almost completely, confirming that the functionalization was successful.

Moreover, TEM micrographs are shown (figure 3*g*,*h*) and, as expected, the size of the dots was not influenced by this treatment. **W-4A** consists of particles of about 15 nm, while **W-4B** shows dots in the range of 7 ± 3 nm. Bigger agglomerates up to 50 nm are visible in both cases.

Also in this case, **W-4A** emits at a slightly longer wavelength than **W-4B**, with *λ*_max_ at 444 nm and 440 nm, respectively (figure 4). Furthermore, the QY and the lifetimes were measured (table 1). As expected, the QYs of around 5% are slightly inferior to the ones of the **W-3** series, but much higher when compared with the non-functionalized CQDs (**W-2** series, QYs less than 1%). Radiative rate constants *k*_r_ of 6.06 × 10^4^ s^−1^ for **W-4A** and 6.53 × 10^4^ s^−1^ for **W-4B** are even smaller than those for **W-3A** and **W-3B**, explaining why the QYs are inferior. Non-radiative rate constants tend to be similar to the results of the previous functionalization, with *k*_nr_ values of 1.06 × 10^6^ s^−1^ for **W-4A** and 1.41 × 10^6^ s^−1^ for **W-4B**.

Finally, the CQDs from the batches **W-4A/B** were dispersed in toluene and put in contact with the non-miscible water phase. The contrary was done in the case of **W-3A/B** CQDs. Both types of dots stayed in their initial phase, even after vigorous shaking (as shown in the inset of figure 4), confirming that polar and apolar CQDs were indeed obtained.

### 3.7. Carbon quantum dots produced using red wine lees as starting material

Red wine lees were also treated with the same procedures. The spectra of the intermediates, **R-1A**, **R-1B**, **R-2A** and **R-2B**, can be found in the electronic supplementary material, figures S4–S8. The resulting CQDs, functionalized with EDA (**R-3A** and **R-3B**), possess on average a comparable photoemission to **W-3A/B**. Furthermore, the wavelengths of excitation and emission are in the same range, at 448 nm for **R-3A** and 443 nm for **R-3B** (electronic supplementary material, figure S9). The QYs and the lifetimes are given in table 1. The values obtained for **R-3A** and **R-3B** are comparable with the ones of **W-3A/B** (around 6%). Also the radiative and non-radiative rate constants are quite similar, with a *k*_r_ of 9.56 × 10^4^ s^−1^ and 1.18 × 10^5^ s^−1^ for **R-3A** and **R-3B**, respectively. The values of *k*_nr_ are 1.64 × 10^6^ s^−1^ and 1.70 × 10^6^ s^−1^, confirming again why the QY is so low. The similarities are also confirmed by IR (electronic supplementary material, figure S10): the surface functionalization of the CQDs is similar compared with that of CQDs from white lees and all the already-discussed signals can be seen on the spectra. This indicates that the method is robust and is not depending on the type of wine lees employed.

Furthermore, TEM micrographs were recorded and also, in this case, no noticeable differences were observed (electronic supplementary material, figure S11). While small isolated particles are visible for **R-3A**, **R-3B** tends to form agglomerates, similar to **W-3B**.

The functionalization with DDA was also performed and the results, **R-4A** and **R-4B**, were again comparable to that for **W-4A/B**. Similar photoemission parameters (*λ*_max_) are observed (447 nm for **R-4A** and 431 nm for **R-4B**), as well as the expected IR signals (same as **W-4A/B**) and the difference in the preferred dispersion media (water versus toluene; electronic supplementary material, figures S9, S12 and S13). The TEM micrographs (electronic supplementary material, figure S11) confirmed that the core size of the dots is not significantly influenced by the post-extraction treatments, though agglomerates of 20–50 nm are observed.

Finally, the QYs and the lifetimes are in the same range as for **W-4A/B** (table 1) and the radiative rate constants (4.85 × 10^4^ s^−1^ for **R-4A** and 6.44 × 10^4^ s^−1^ for **R-4B**) are again much smaller than the non-radiative rate constants (1.30 × 10^6^ s^−1^ for **R-4A** and 1.42 × 10^6^ s^−1^ for **R-4B**).

### 3.8. Ink-jet printing of carbon quantum dots

**Figure 5.**
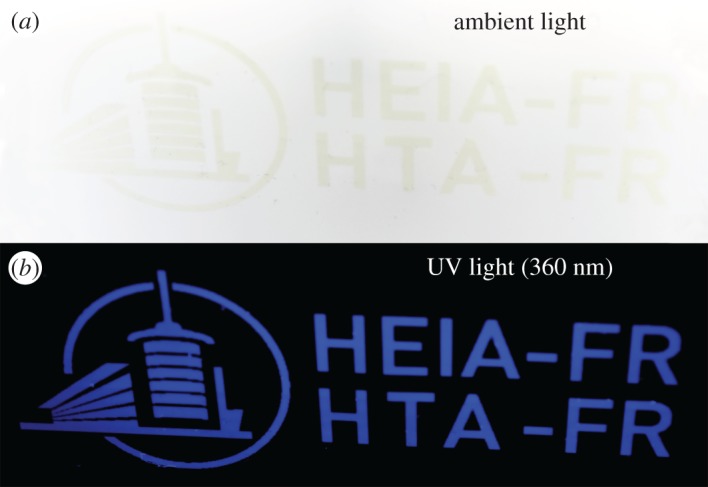
Ink-jet deposition of **R-4A** dispersed in a UV curable varnish (*a*) in ambient light and (*b*) under UV light (360 nm).

The CQDs suspended in an acrylic base were printed. They are almost invisible in ambient light but yield an intense blue when irradiated at 360 nm, as shown in figure 5. The high-resolution printing (1200 dpi) ran smoothly without printhead clogging, confirming that CQDs are suitable pigments for ‘invisible’ inks. Even in the case of agglomeration, the danger of clogging the nozzle is much lower when compared with the usual pigments whose initial dimensions are of a few hundreds of nanometres.

## 4. Conclusion

Using the wine lees as a row material for producing CQDs is a very interesting way to revalorize this important by-product of wine fermentation. It is possible to obtain CQDs in a very easy and low-cost way just by extracting them from the carbonized wine lees that already contain all the ingredients to produce exploitable CQDs. Moreover, this method gives similar results when different starting materials (white or red wine lees) are used. We have also demonstrated the possibility to tailor some of their features by post-extraction treatment in order to fine-tune their properties (better QY or dispersible in different solvents), by applying simple and well-known chemical reactions. Hence it can be inferred as a non-problematic scale-up.

We were able to obtain CQDs with a QY of around 6%, which is a common value for pure CQDs according to the literature [45], as well as CQDs dispersible in organic solvents, which is still quite rare in the literature [46]. CQDs are among the most promising multifunctional materials of the future; thus with this contribution, we aim to help establish a complete and reliable synthetic pathway for different applications.

## Supplementary Material

Synthesis of hydrophilic or hydrophobic carbon quantum dots from waste of wine fermentation

## Data accessibility

Electronic supplementary material can be found online on the website of the publisher.
