# Supplementary material for: Synthesis of hydrophilic and hydrophobic carbon quantum dots from waste of wine fermentation
Source: R Soc Open Sci. 2017 Dec 20;4(12):170900. doi: 10.1098/rsos.170900 (PMC5749999; doi:10.1098/rsos.170900)
Supplement: Synthesis of hydrophilic or hydrophobic carbon quantum dots from waste of wine fermentation [file rsos170900supp1.docx]

**Electronic Supplementary Information**

**Synthesis of hydrophilic or hydrophobic carbon quantum dots from waste of wine fermentation**

Massimo Varisco^a+^, Denis Zufferey^a+^, Albert Ruggi^b^, Yucheng Zhang^c^, Rolf Erni^c^ and Olimpia Mamula^a*^

Table of Contents

SI Table 1 Summary of solvents studied during the extraction optimisation Page 3

SI Figure 1 Emission spectra at different concentrations Page 4

SI Figure 2 Emission spectra of CQDs extracted with different solvents Page 5

SI Figure 3 Emission spectra of the **W-1** and **W-2** series Page 6

SI Figure 4 Absorbance spectra of **W-3A**, **W-3B**, **R-3A** and **R-3B** Page 7

SI Figure 5 Emission spectra of the **R-1** and **R-2** series Page 8

SI Figure 6 IR spectra of the **R-1** series Page 9

SI Figure 7 TEM micrographs of the **R-1** and **R-2** series Page 10

SI Figure 8 IR spectra of the **R-2** series Page 11

SI Figure 9 Emission spectra of the **R-3** and **R-4** series Page 12

SI Figure 10 IR spectra of the **R-3** series Page 13

SI Figure 11 TEM micrographs of the **R-3** and **R-4** series Page 14

SI Figure 12 IR spectra of the **R-4** series Page 15

SI Figure 13 Picture of the **R-3** and **R-4** series dispersed in polar and Page 16

apolar media

SI Figure 14 Absorbance spectra of **W-1A**, **W-1B**, **R-1A** and **R-1B** Page 17

SI Figure 15 Absorbance spectra of **W-2A**, **W-2B**, **R-2A** and **R-2B** Page 18

SI Figure 16 Absorbance spectra of **W-4A**, **W-4B**, **R-4A** and **R-4B** Page 19

SI Figure 17 Emission spectra of **R3-B** excited at different wavelengths Page 20

| Solvent | λ_max_ [nm] for a 0.2 g / L solution | Yield |
| --- | --- | --- |
| Water | 440 | 65% |
| Methanol | 445 | 55% |
| Ethanol | 440 | 60% |
| Isopropanol | 435 | 5.5% |
| Ether | 438 | 5% |
| Toluene | 432 | 2.5% |
| *n*-heptane | -- | 31% |
| cyclohexane | -- | 7% |

SI Table 1: Summary of the solvents used for the extraction of CQDs from carbonised lees, their maximum absorbance and extraction yields.


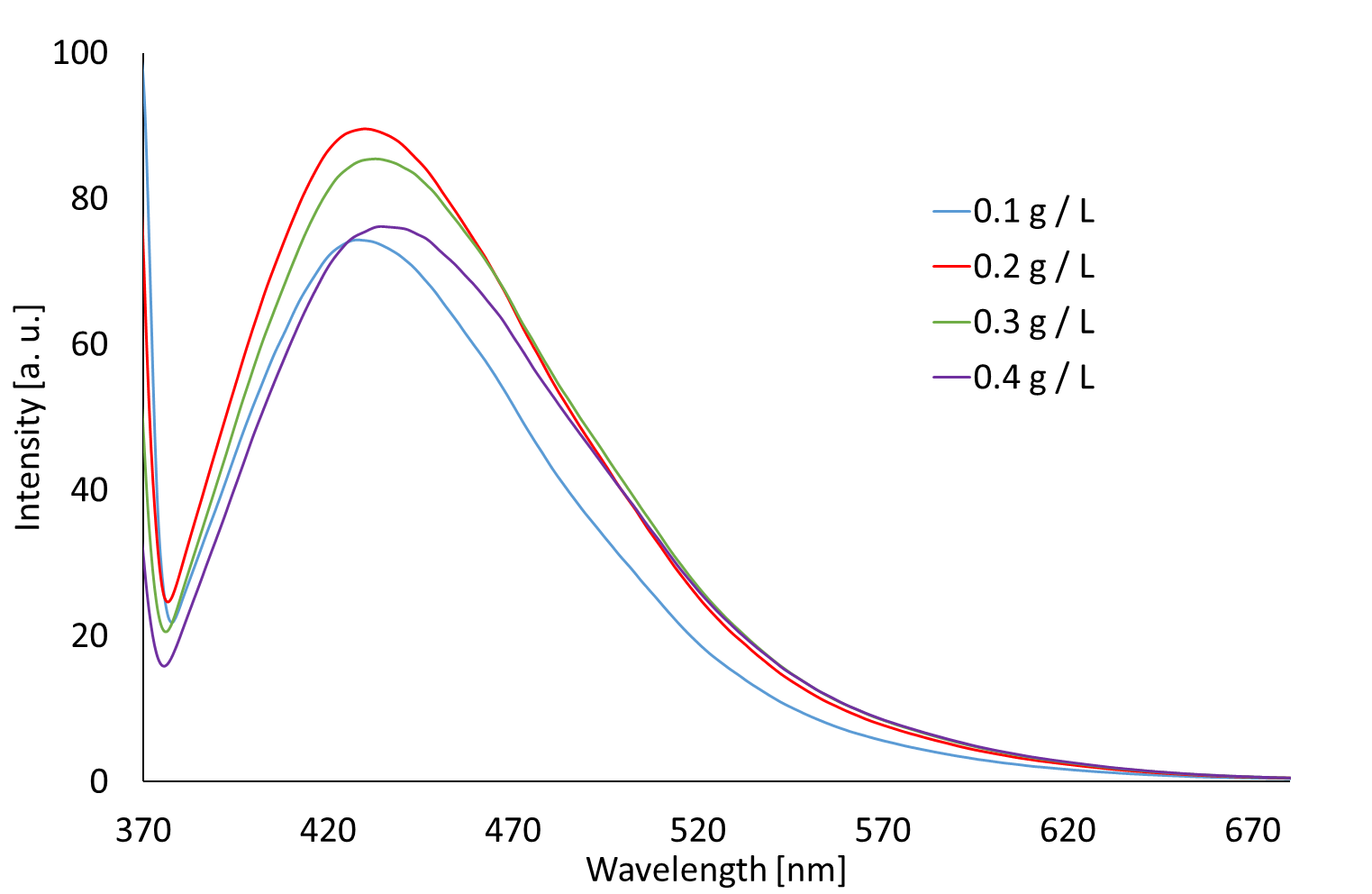


SI Figure 1: Emission of CQDs at different concentrations. Excitation at 360 nm.


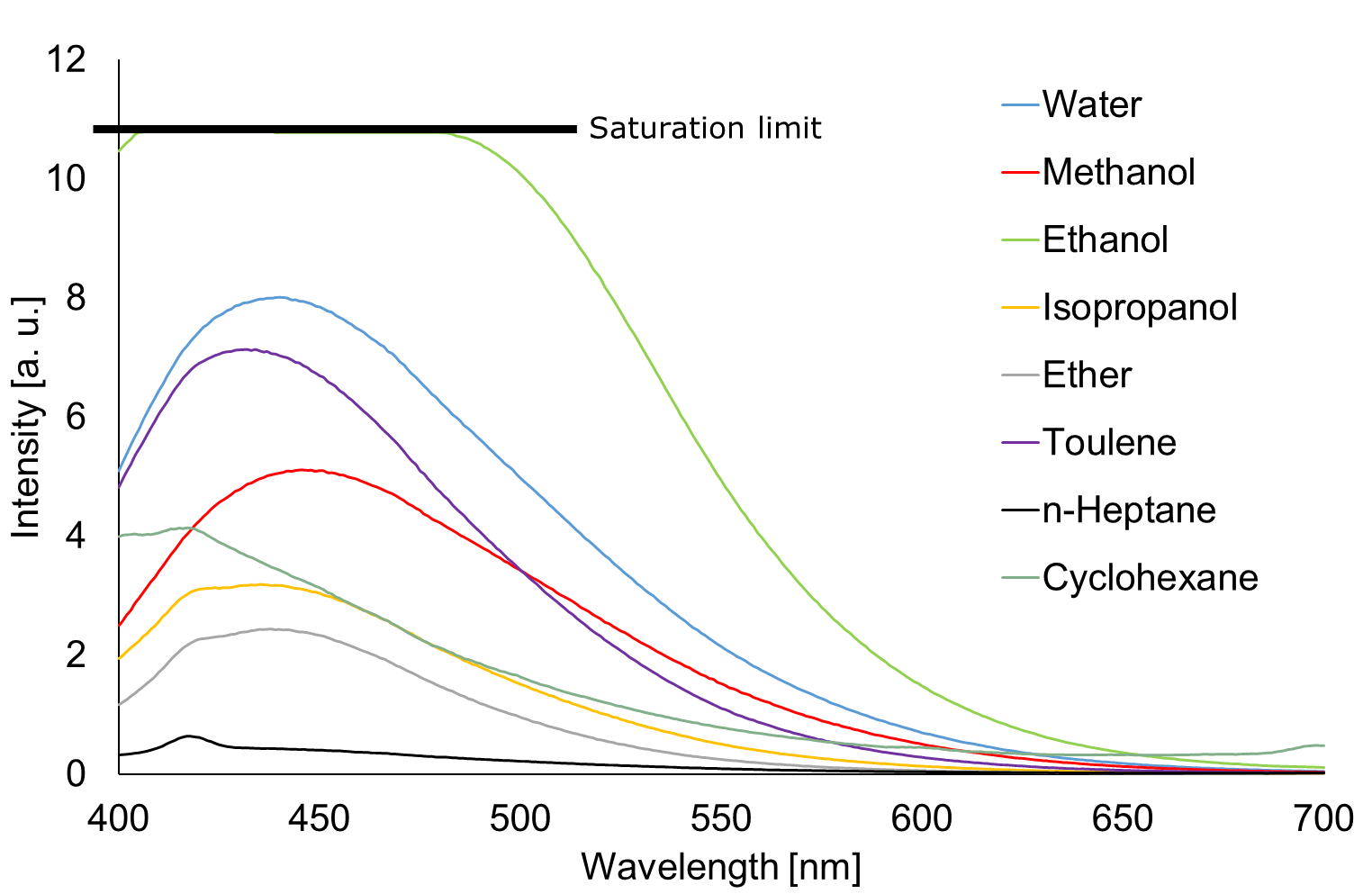


SI Figure 2: Emission spectra (excitation at 360 nm, concentration 0.2 g / L in deionized water) of CQDs extracted with different solvents from the carbonised lees.


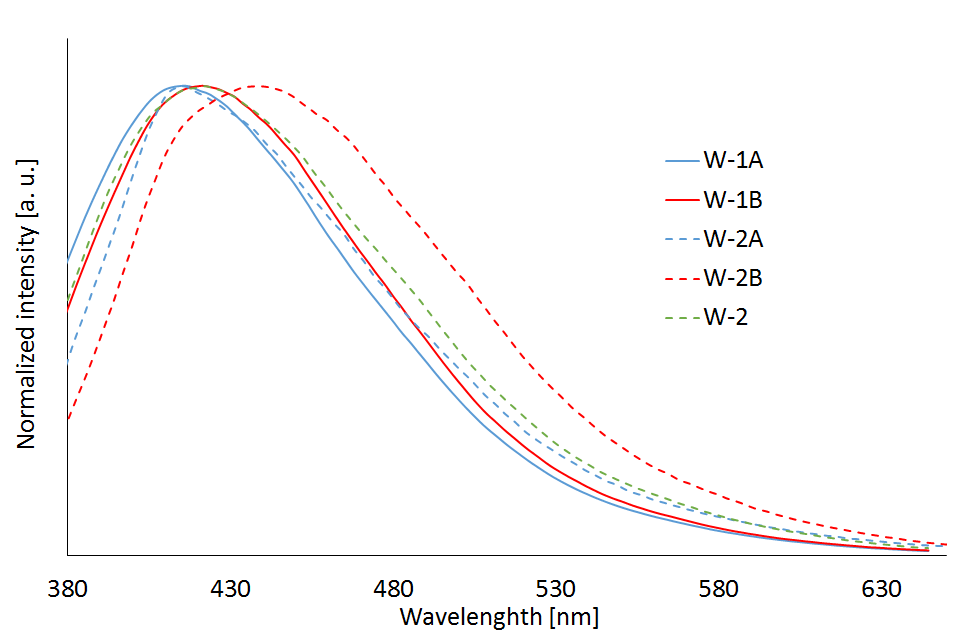


SI Figure 3: Emission spectra (excitation at 360 nm, concentration 0.2 g / L in deionized water) of **W-1A** (solid blue line), **W-1B** (solid red line), **W-2A** (dashed blue line), **W-2B** (dashed red line) and **W-2** (dashed green line).


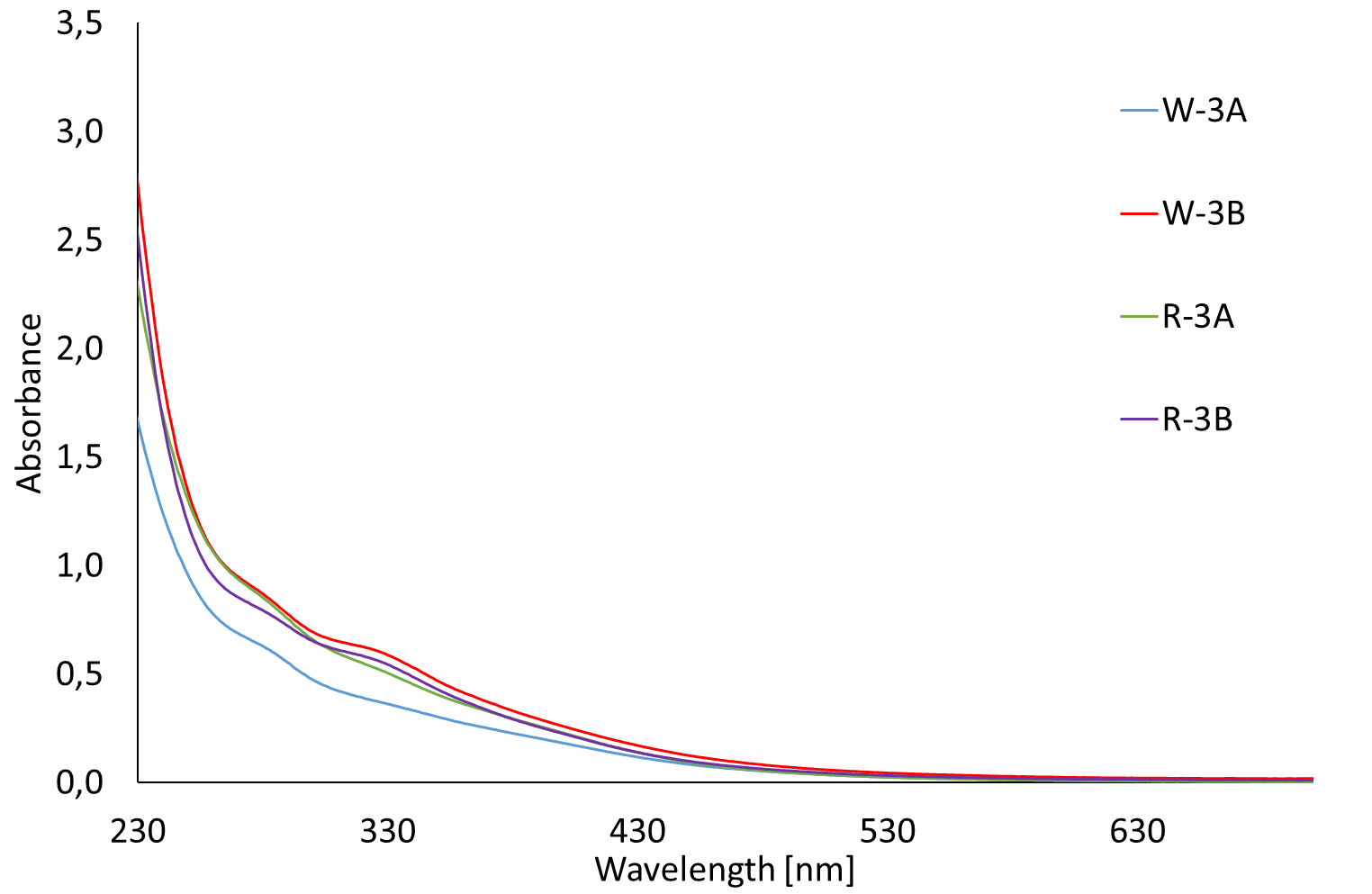


SI Figure 4: UV/Vis absorbance spectra (0.2 g / L in deionised water) of **W-3A** (blue line), **W-3B** (red line), **R-3A** (green line) and **R-3B** (purple line).


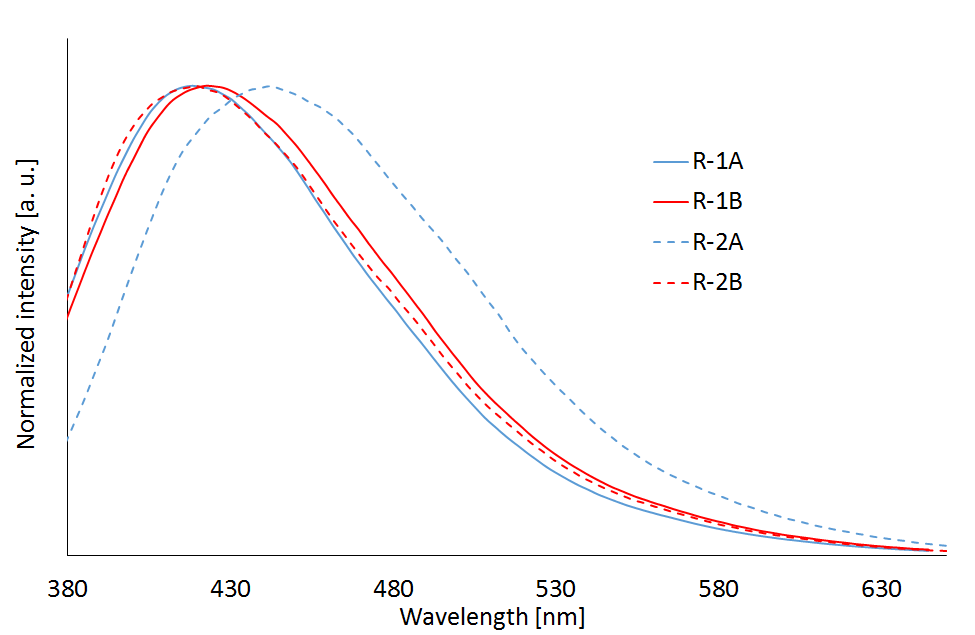


SI Figure 5: Emission spectra (excitation at 360 nm, concentration 0.2 g / L in deionized water) of **R-1A** (solid blue line), **R-1B** (solid red line), **R-2A** (dashed blue line) and **R-2B** (dashed red line).


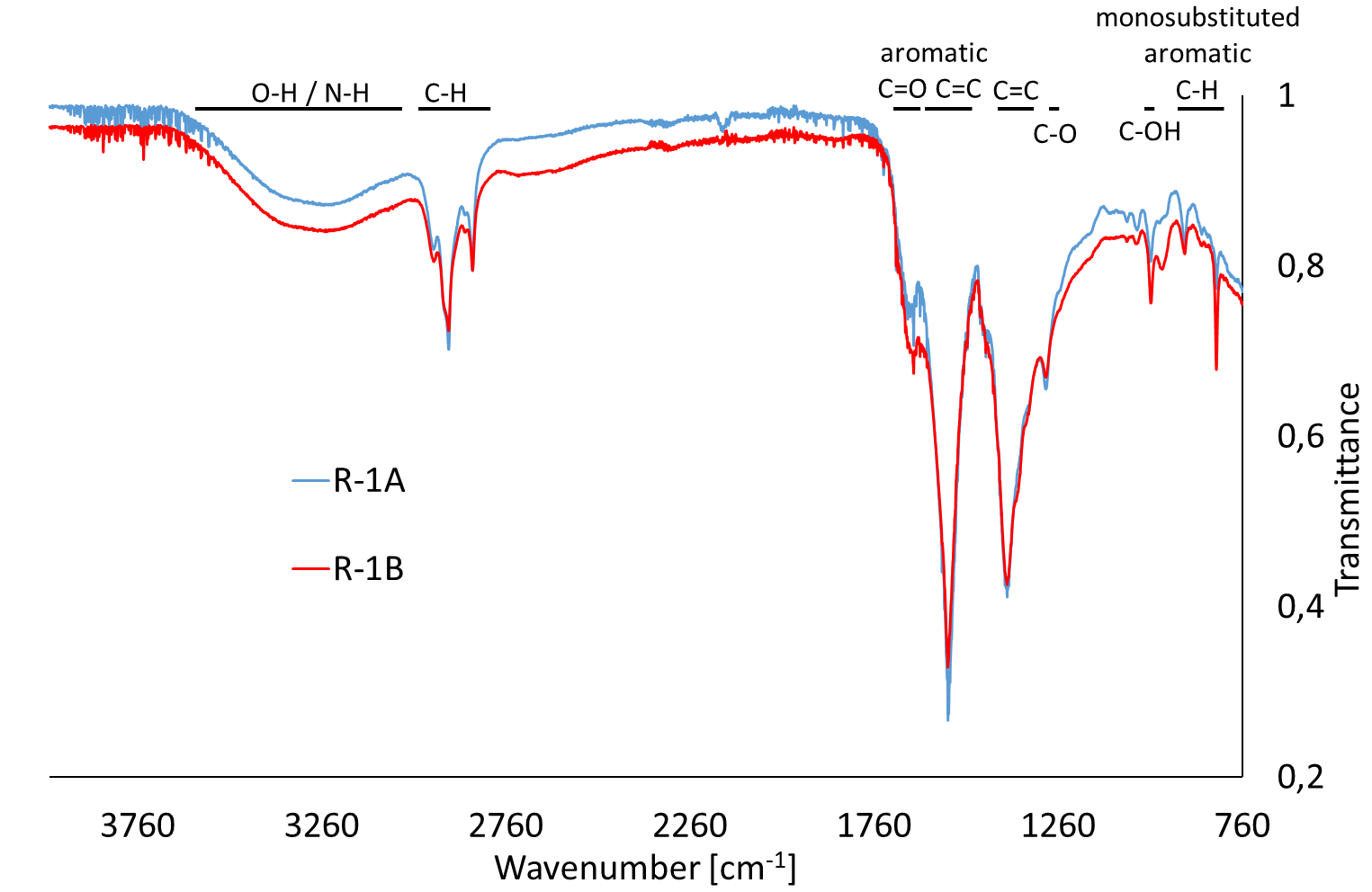


SI Figure 6: IR spectra of **R-1A** (blue line) and **R-1B** (red line).


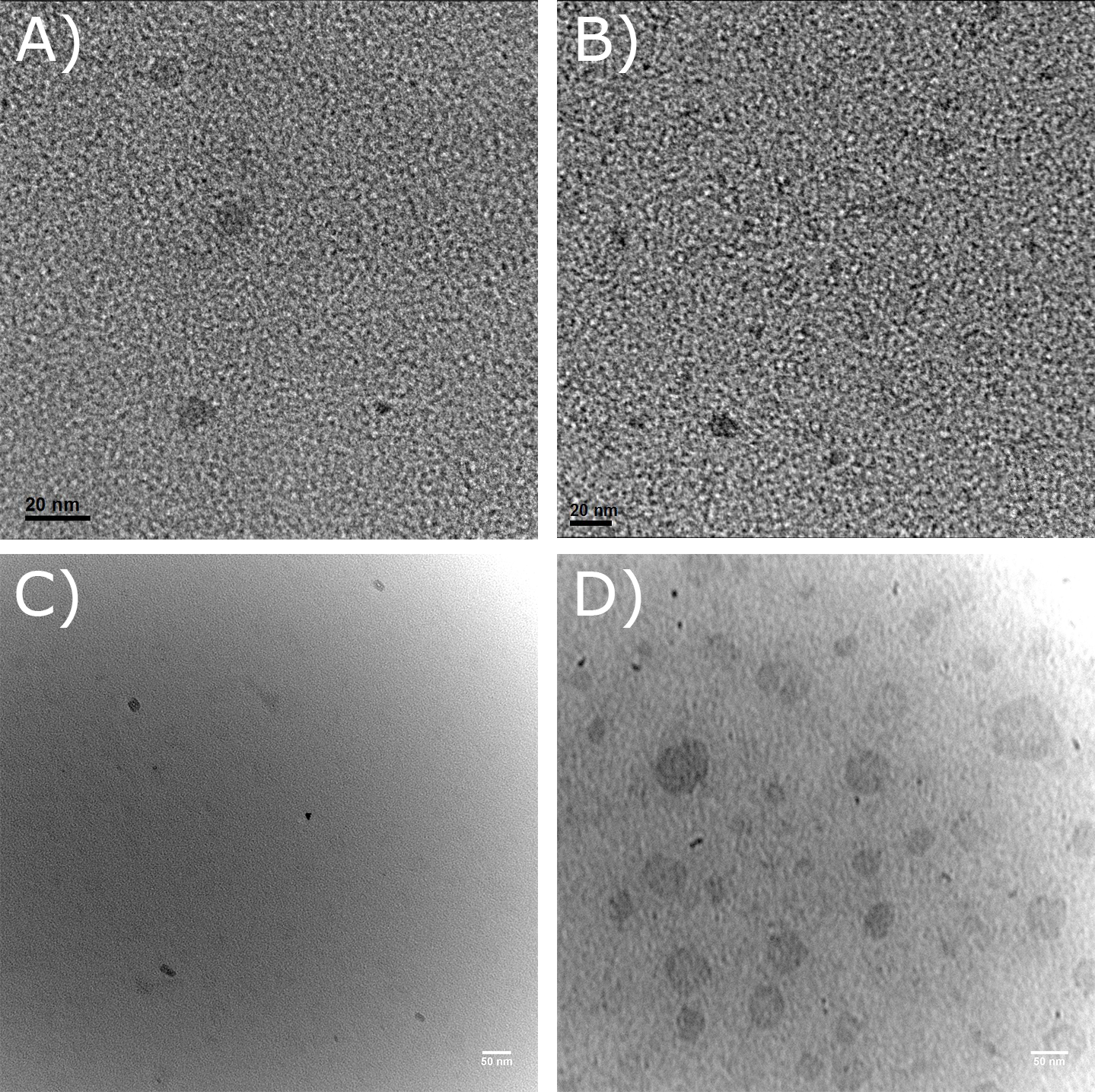


SI Figure 7: TEM micrographs of A) **R-1A**. B) **R-1B**. C) **R-2A**. D) **R-2B**.


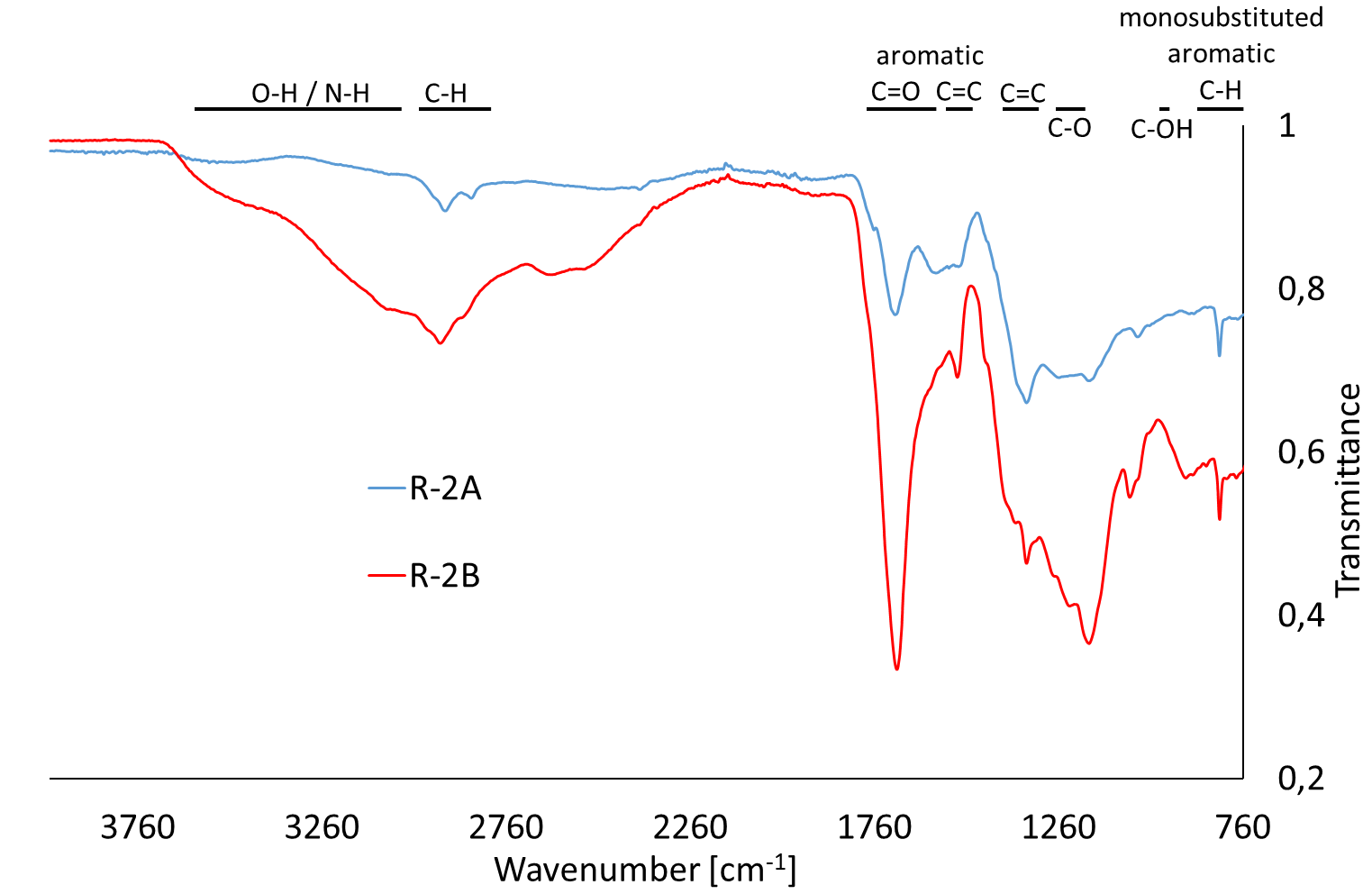
SI Figure 8: IR spectra of **R-2A** (blue line) and **R-2B** (red line).


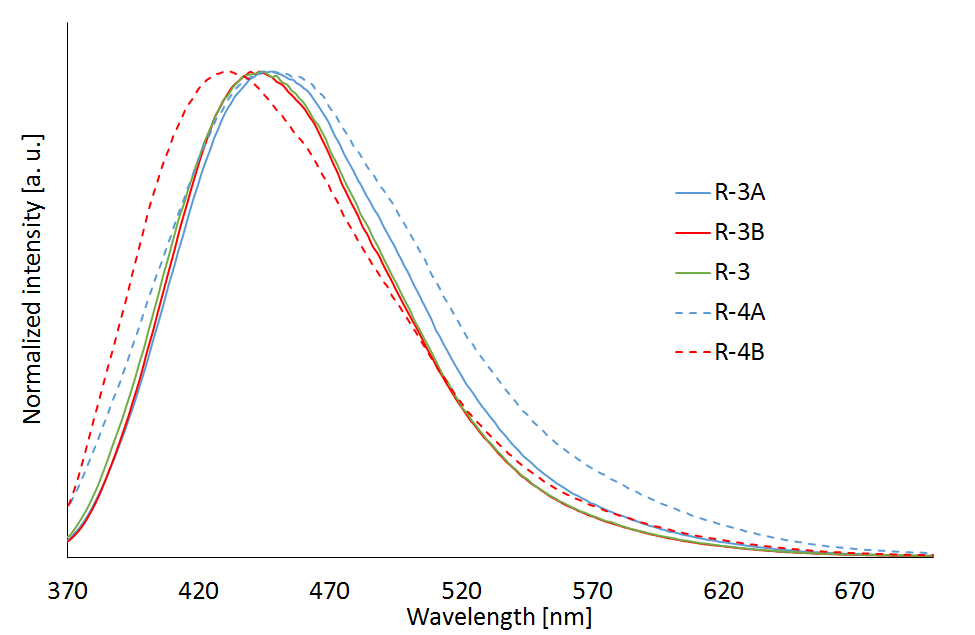


SI Figure 9: Emission spectra (excitation at 360 nm, concentration 0.2 g / L in deionised water for the **R-3** series and concentration 0.2 g / L in toluene for the **R-4** series) of **R-3A** (solid blue line), **R-3B** (solid red line), **R-3** (solid green line), **R-4A** (dashed blue line) and **R-4B** (dashed red line).


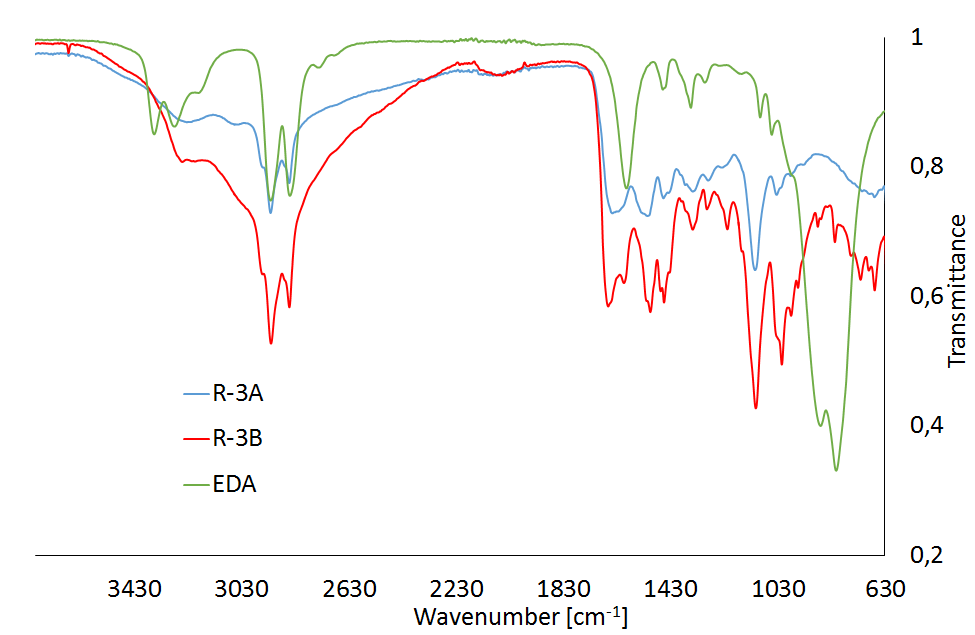


SI Figure 10: IR spectra of **R-3A** (blue line), **R-3B** (red line) and EDA as reference (green line).


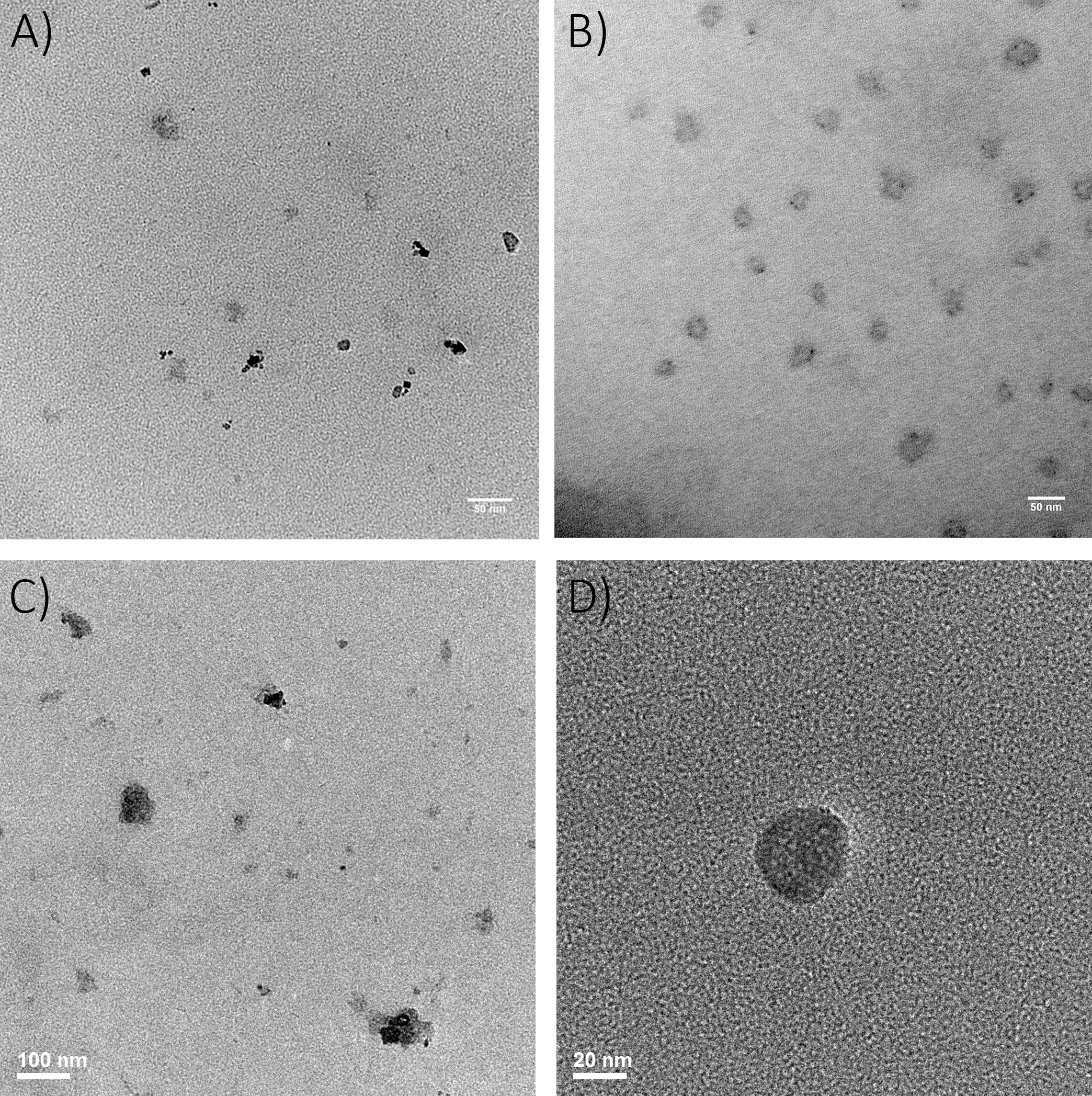


SI Figure 11: TEM micrographs of A) **R-3A**. B) **R-3B**. C) **R-4A**. D) **R-4B**.


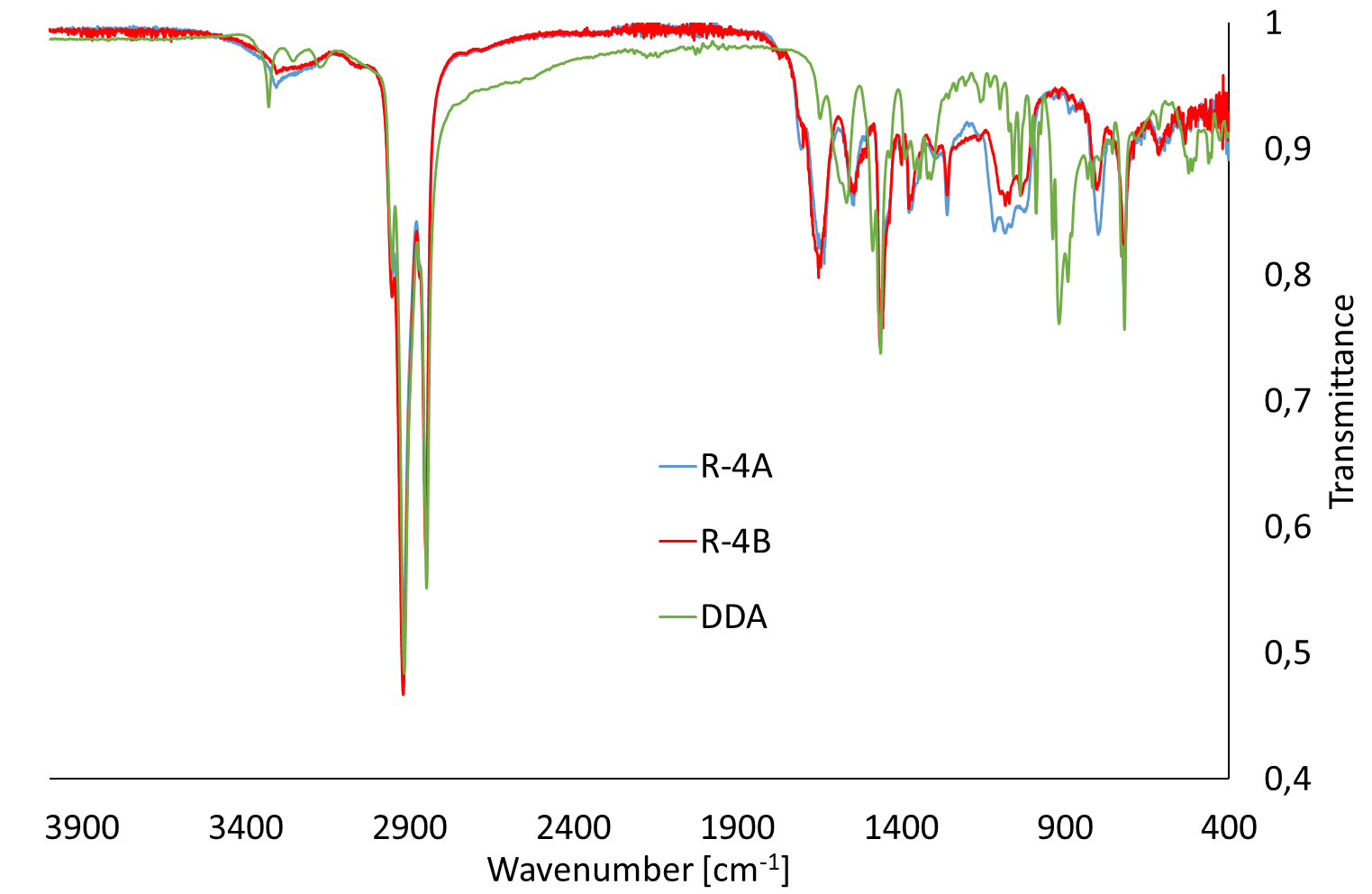


SI Figure 12: IR spectra of **R-4A** (blue line), **R-4B** (red line) and DDA (green line) as reference.


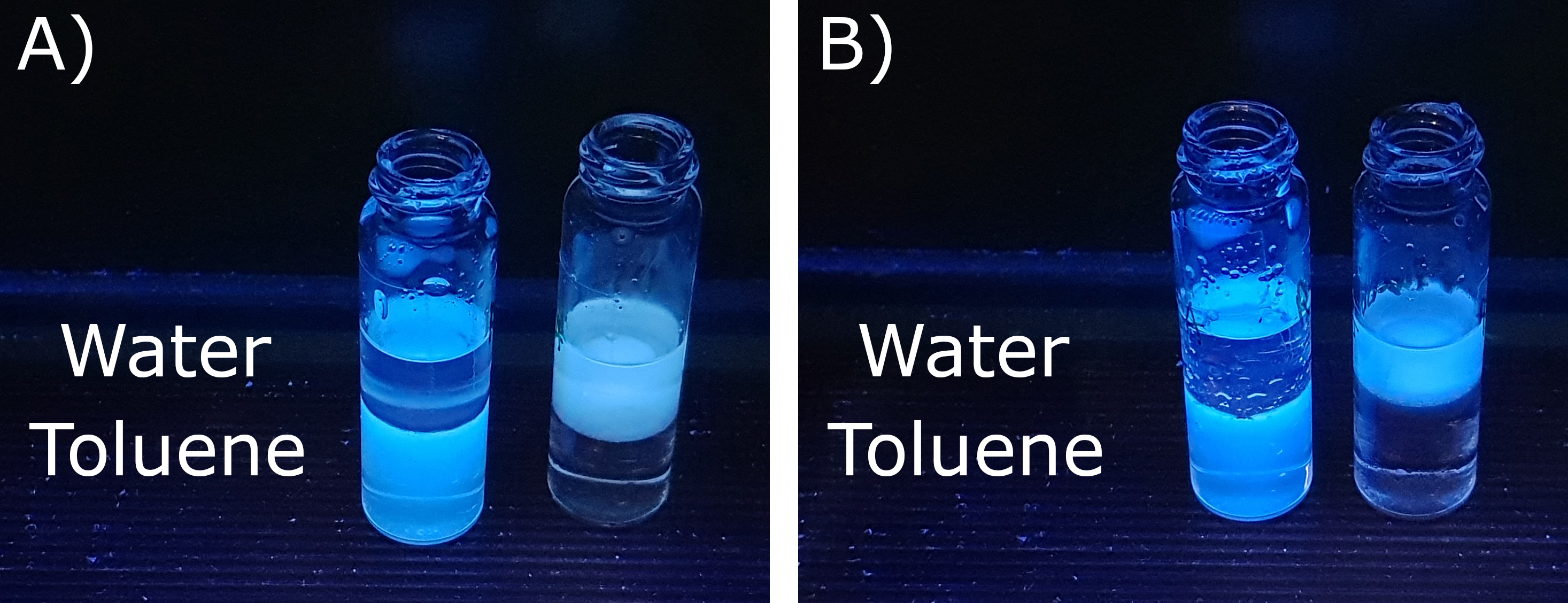


SI Figure 13: pictures of the following samples, under excitation at 366 nm: A) **R-4A** dispersed in toluene (left) and **R-3A** dispersed in water (right); B) **R-4B** dispersed in toluene (left) and **R-3B** dispersed in water (right).


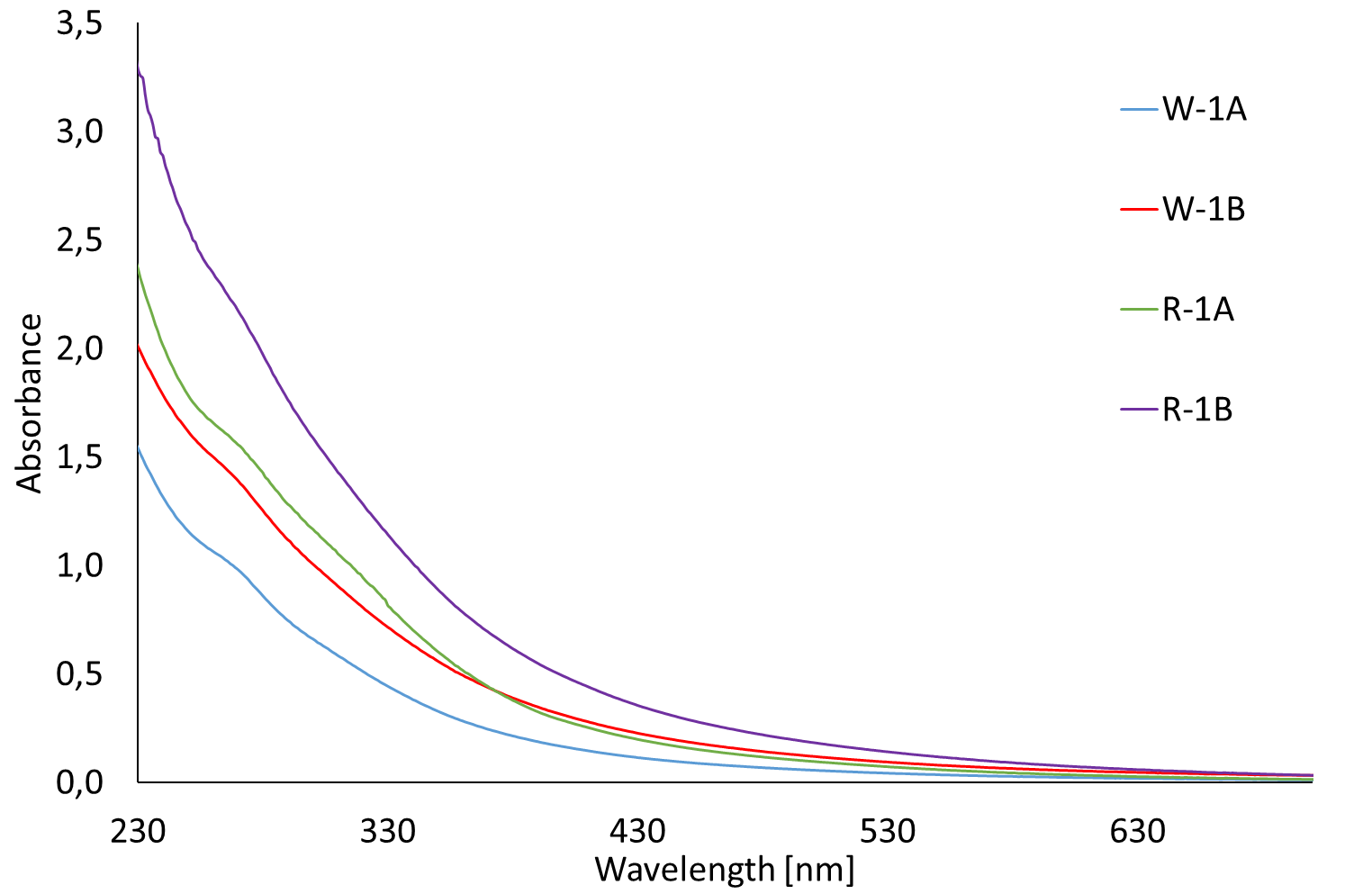


SI Figure 14: UV/Vis absorbance spectra (0.2 g / L in deionised water) of **W-1A** (blue line), **W-1B** (red line), **R-1A** (green line) and **R-1B** (purple line).


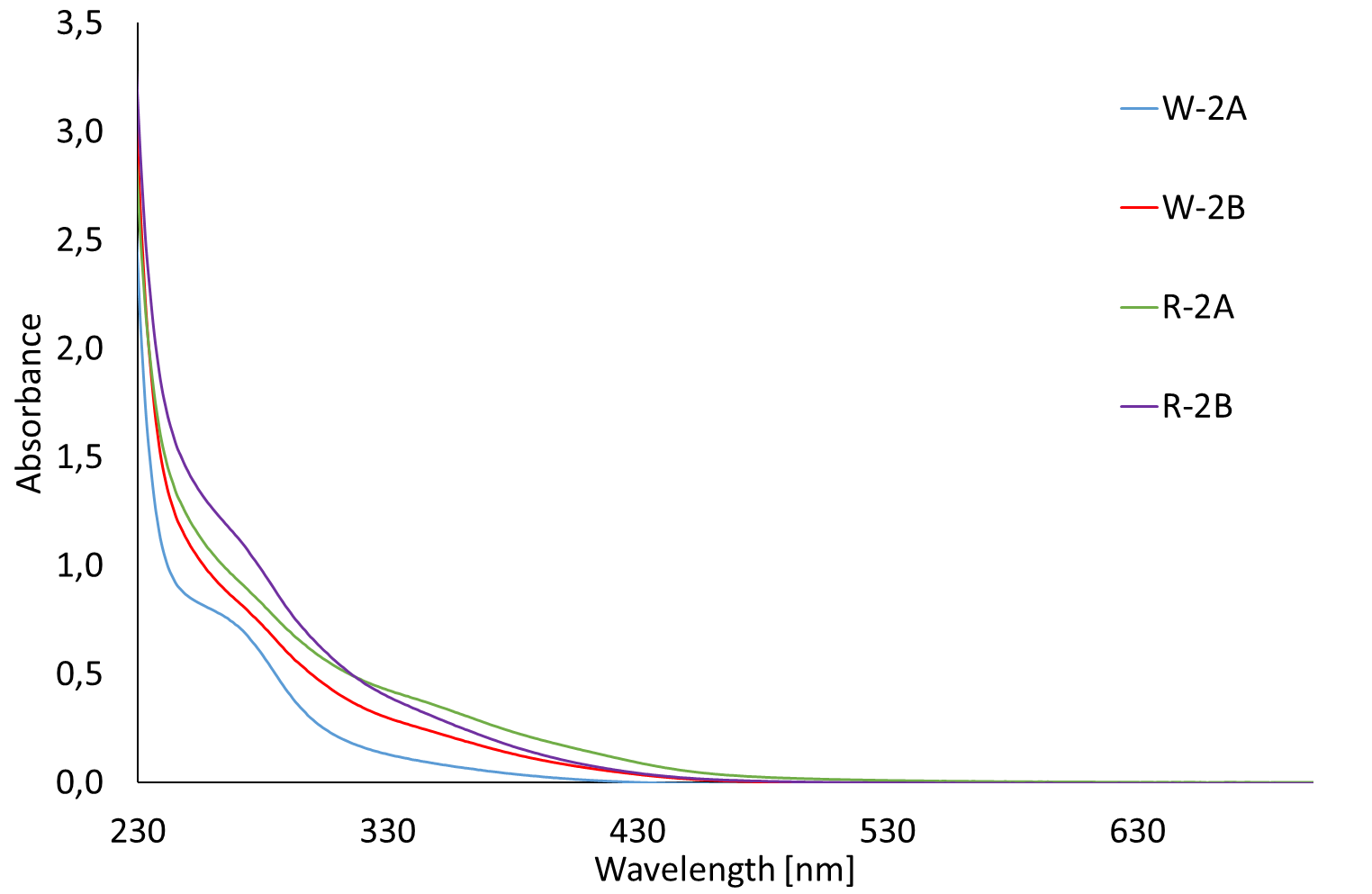


SI Figure 15: UV/Vis absorbance spectra (0.2 g / L in deionised water) of **W-2A** (blue line), **W-2B** (red line), **R-2A** (green line) and **R-2B** (purple line).


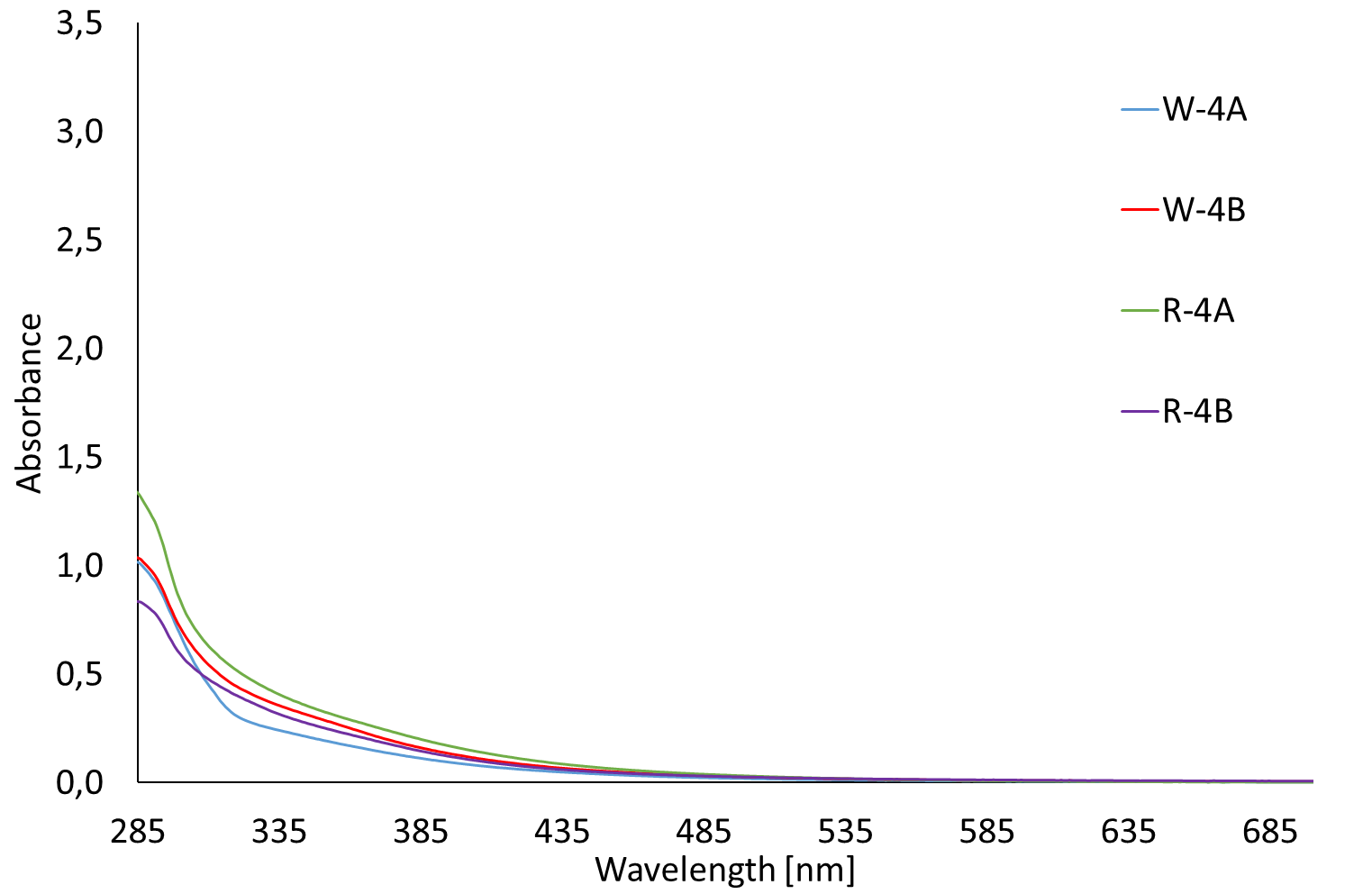


SI Figure 16: UV/Vis absorbance spectra (0.2 g / L in toluene) of **W-4A** (blue line), **W-4B** (red line), **R-4A** (green line) and **R-4B** (purple line).


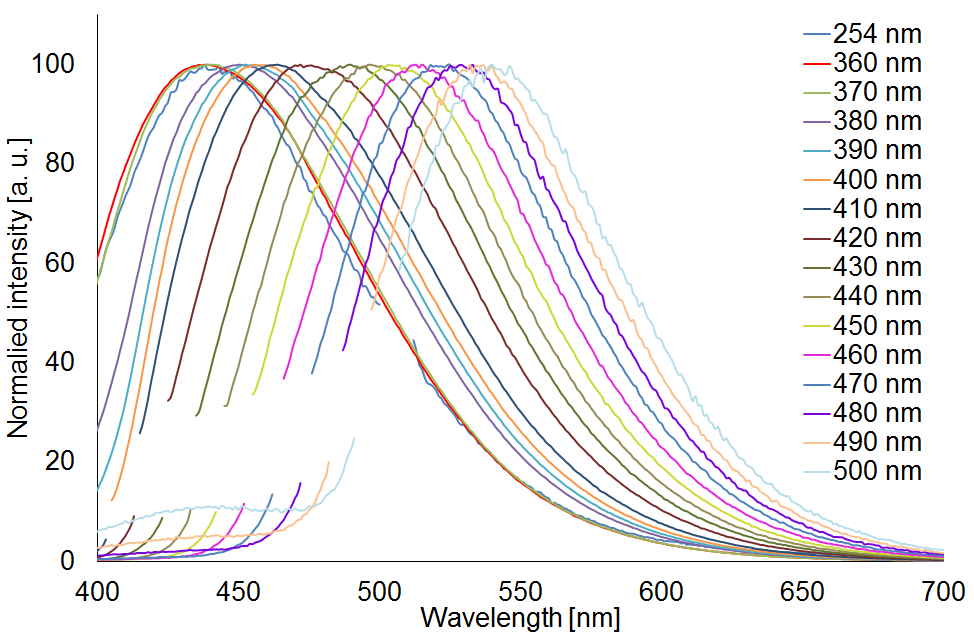


Si Figure 17: tipical emission spectra (0.2 g / L in deionised water) of CQDs excited at different wavelengths. Case studied: **R-3B**.
